# Roles of GLUT-1 and HK-II expression in the biological behavior of head and neck cancer

**DOI:** 10.18632/oncotarget.24684

**Published:** 2019-04-30

**Authors:** Hang Yang, Jiang-Tao Zhong, Shui-Hong Zhou, He-Ming Han

**Affiliations:** ^1^ Department of Otorhinolaryngology, The First Affiliated Hospital, College of Medicine, Zhejiang University, Hangzhou, Zhejiang, 310003, China; ^2^ Present Address: Department of Otorhinolaryngology, The People’s Hospital of Jiangshan City, Jiangshan, Zhejiang, 324100, China

**Keywords:** glucose transporter-1, hexokinase II, Warburg effect, head and neck cancer

## Abstract

The Warburg effect plays an important role in the proliferation and invasion of malignant tumors. Glucose transporter 1 and hexokinase II are two key energy transporters involved in mediating the Warburg effect. This review will analyze the mechanisms of these two markers in their effects on the biological behavior of head and neck cancer.

## INTRODUCTION

Carcinogenesis is a multistep process [1], in which abnormal glucose metabolism may play an important role [2, 3]. Abnormal energy metabolism of malignant tumors is a subject of current research. Glucose provides mammalian cells with their main source of energy, via glucose transporters (GLUTs) expressed on the cell membrane, which mediate glucose entry into cells. Energy is accessible to cells via two pathways: mitochondrial oxidative phosphorylation and glycolysis. The first key enzyme involved in glycolysis is a member of the hexokinase (HK) family. Thus, GLUTs and HKs are important enzymes mediating glucose metabolism in tumorigenesis [4]. Glycolysis provides only 10% of the energy in normal cells and relatively few of the total ATP molecules (2 ATP) generated per molecule of glucose broken down, compared with aerobic respiration, which produces 38 ATP molecules. Rapid proliferation of malignant tumors requires high levels of energy; therefore, tumors must accelerate glucose uptake and increase their rate of glycolysis [5]. The use of glycolysis by malignant cells to generate energy, whether in an aerobic or anaerobic environment, is known as the Warburg effect. However, the precise mechanism of this effect is not clear. It may involve the interaction of many factors, such as abundant lactic acid production during glycolysis, the escape of malignant tumors from immune surveillance, prevention of apoptosis, etc. Studies have found that members of the GLUT family, GLUT-1 in particular, and of the HK family, especially HK-II, are associated with the transport of glucose and mediation of glucose metabolism. Abnormal expression of GLUT-1 and HK-II in malignant tumors is associated with invasion and metastasis of tumors including head and neck cancers [6]. The tumor microenvironment is hypoxic, which is related to abnormal expression of GLUT-1 and HK-II. Many studies have shown that inhibition of GLUT-1 and HK-II expression aberrations can improve the treatment efficacy of malignant tumors [7–9]. Thus, inhibition of GLUT-1 and HK-II may represent a novel treatment strategy for head and neck cancers.

### GLUT-1

#### Research history of GLUT-1

The study of transmembrane glucose transport dates back approximately 100 years. In 1977, GLUT-1 was first isolated from red blood cells [10]. In 2014, Yan et al. were the first to analyze the crystal structure of human GLUT-1 [11].

#### Biological characteristics of GLUT-1

Glucose is the primary source of cellular energy. The first step in glucose metabolism is glucose entry into the cell, a process that relies on glucose transport proteins. GLUTs, of which GLUT-1 was the first discovered and is the most widely distributed, are embedded in the cell membrane to transport extracellular glucose into the cell. Reports have found that GLUTs consist of 12 transmembrane protein domains. GLUTs exist in almost every cell in the body and are important not only for maintenance of normal human body functions but also tumor metabolism [12]. Tumor cells need to consume excess glucose to maintain growth and proliferation. GLUT-1 transports glucose into cells without requiring energy. GLUT-1 is expressed at low levels in normal tissues and benign lesions, whereas high expression is often correlated with carcinogenesis and can indicate a poor prognosis or recurrence [5].

#### GLUT-1 and malignant tumors

#### GLUT-1 and development of malignant tumors

GLUT-1 may play an important role in carcinogenesis. High GLUT-1 expression has been reported in colorectal carcinoma [13], lung carcinoma [14], breast carcinoma [15], esophageal carcinoma [16], gastric carcinoma [17], ovarian carcinoma [18], cholangiocarcinoma [19], and head and neck squamous cell carcinoma [20]. In malignant melanoma, Koch et al. found that GLUT-1 expression increased correspondingly with tumor progression from nevus (early stage) to advanced stages. They reported that inhibition of GLUT-1 expression can reduce the growth, apoptosis, and migration of malignant melanoma [21]. Lu et al. reported positive GLUT-1 expression in 73.6% of 53 pancreatic cancer samples, which was significantly higher than that in a control group [22]. According to one study, expression of GLUT-1 in rectal cancer was 93.6% [23]. GLUT-1 expression in hyperplasia, endometrial carcinoma, type I endometrial carcinoma, and type II endometrial carcinoma was 88.9%, 98.5%, 98% and 100%, respectively. GLUT-1 expression was also associated with cancer grade in endometrial carcinoma [24].

#### GLUT-1 and tumor staging

Lu et al. found that high expression of GLUT-1 was associated with the clinical stage of pancreatic cancer and with the standard uptake value (SUV) and Ki67 expression [22]. In endometrial carcinoma, the expression of GLUT-1 was related to SUV and was significantly higher in the FIGO stage IB and IC phase than the IA phase (*P*=0.001, *P*=0.003) [25].

A study examining the expression of hypoxia inducible factor-1α (HIF-1α), carbonic anhydrase-IX (CA-IX), GLUT-1, and vascular endothelial growth factor (VEGF) in 54 advanced cervical cancers reported that GLUT-1 expression was associated with tumor stage and lymphovascular involvement [26].

However, controversy remains regarding the relationship between GLUT-1 expression and the tumor stage. A study that measured the expression of GLUT-1, GLUT-3, GLUT-6, and GLUT-10 in 150 gastric carcinomas by immunohistochemical (IHC) methods found that GLUT-1 was not associated with tumor stage or prognosis, but that GLUT-3 was associated with tumor stage (per the Union for International Cancer Control (UICC) guidelines) and survival [27]. In endometrial cancer, Sadlecki et al. found that neither GLUT-1 nor CA-IX was associated with the FIGO clinical stage, histological grade, Bokhman subtypes, lymph node involvement, distant metastases, deep myometrial invasion, or recurrence [28].

#### GLUT-1 and metastasis of malignant tumors

Koch et al. found that GLUT-1 was expressed more strongly in melanoma metastases than in primary melanomas [21]. In pancreatic neuroendocrine tumors, GLUT-1 expression in lymph node metastases was significantly higher than that in non-metastatic tumors [29]. High expression levels of GLUT-1 in cervical cancer [26], ovarian cancer [30], gastric cancer [31], and non-small-cell lung cancer [32] have been shown to be related to lymph node metastasis [30].

#### GLUT-1 and prognosis of malignant tumors

Several studies have reported that GLUT-1 is a prognostic factor in malignant tumors [22, 33, 34]. In a study of colorectal cancer, Lee et al. found that the SUV and retention index (RI) (a high RI score predicts poor prognosis) were significantly higher in specimens with high GLUT-1 expression than in those with low GLUT-1 expression [34]. Younes et al. found that the prognosis of patients with bladder cancer was worse for those with greater than 10% compared with less than 10% tumor positivity for GLUT-1 expression (*P*=0.0064) [33]. In pancreatic cancer, the expression of GLUT-1 was associated with not only tumor size, stage, and lymph node involvement but also tumor proliferation and a worse prognosis. Univariate analysis showed that the survival of patients with high GLUT-1 expression was 12.3 months, which was significantly shorter than that of those with low expression (22.2 months), suggesting that high GLUT-1 expression in pancreatic cancer predicts poor prognosis. Multivariate analysis showed that GLUT-1 was the only factor measured that was associated with poor prognosis in pancreatic cancer [22]. Several other studies yielded similar results for pancreatic cancer [35–37]. A meta-analysis of a selection of biomarkers including GLUT-1 found an association of GLUT-1 with poor prognosis in biliary tract cancers [38]. GLUT-1 expression has also been associated with the prognosis of many tumor types, including gastric cancer [39], osteosarcoma [40], meningioma [41], esophageal cancer [42], cervical cancer [43], endometrial cancer [28], and ovarian cancer [30].

#### The relationship between GLUT-1 and other factors

HIF-1α and GLUT-1 are important markers of hypoxia in the cancer microenvironment. GLUT-1 expression is influenced by HIF-1α [44], as well as by mitochondrial oxidative phosphorylation [45]. GLUT-1 and CA-IX have been coined “endogenous hypoxia markers” in some scholarly reports [26]. Studies have shown that the expression of GLUT-1 is affected by many factors, such as glucose deprivation, cellular carcinogenesis, and osmotic pressure [46]. While some studies have found a relationship between GLUT-1 and hypoxia [26, 44–46], others have not found a direct correlation [47, 48]. This divergence may be related to difficulties in identifying hypoxic regions of tissue when examining gangrenous versus living tissue by Polarography observation; the hypoxic regions identified may be inaccurately high [49]. One study showed that GLUT-1 expression is related to Ki-67 expression, a marker of cell proliferation, which is closely related to tumor differentiation, invasion, metastasis, and prognosis [22].

#### Possible mechanisms of resistance to cancer radiotherapy and chemotherapy mediated by GLUT-1

**Figure 1:**
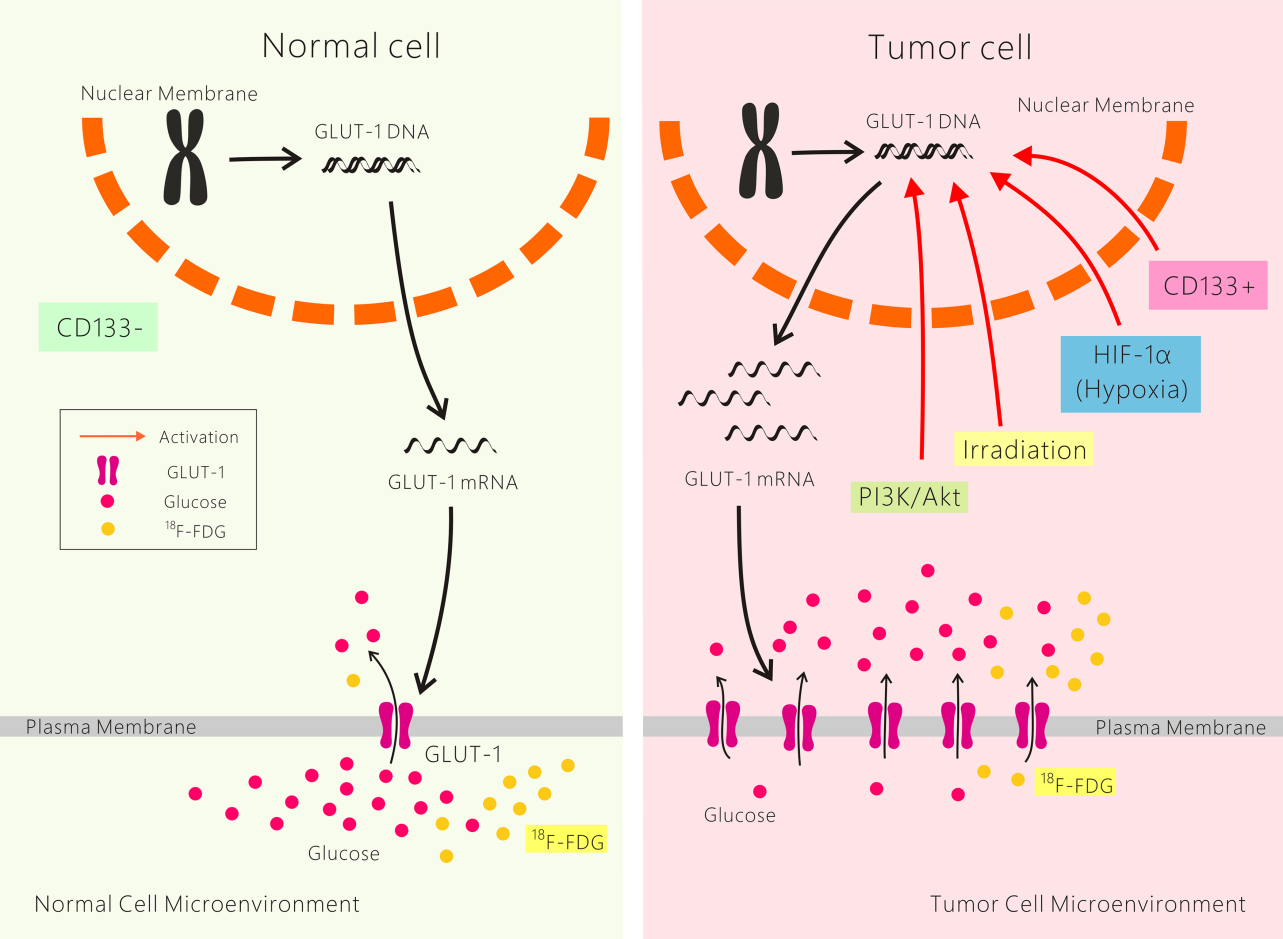
Possible mechanisms of resistance to cancer radiotherapy and chemotherapy mediated by GLUT-1.

The underlying mechanism of GLUT-1 expression, which induces chemo-radioresistance in cancer cells, remains unclear. It is likely caused by interplay among multiple factors. First, the higher energy metabolic rate of malignant tumor cells compared with their nonmalignant counterparts, even in aerobic glycolysis, is called the Warburg effect. Increased GLUT-1 expression plays an important role in cancer glucose metabolism. We suggest that GLUT-1 overexpression results in satisfying the higher energy requirement of cancer and leads to chemo-radioresistance. Second, hypoxia is a common phenomenon in solid tumors. GLUT-1 is a potential intrinsic marker of hypoxia in cancer, and HIF-1α regulates the expression of several hypoxia-response genes, including GLUT-1. As mentioned above, HIF-1α expression is associated with GLUT-1 expression in many cancers, and both markers are associated with the poor prognosis of many cancers. We suggest that GLUT-1 expression is associated with chemo-radioresistance via HIF-1α regulation. Third, GLUT-1 expression is also regulated by the PI3K/Akt pathway. This pathway plays a role in chemo-radioresistance/intrinsic resistance, tumor cell proliferation and hypoxia. The results of our previous and others’ studies suggest that activation of the PI3K/Akt pathway plays a role in GLUT-1-mediated chemo-radioresistance in solid cancers, including laryngeal carcinoma. Fourth, CD133^+^ cancer stem cells may be involved in chemo-radioresistance caused by GLUT-1. In our previous study, we found that chemo-radioresistance in CD133+ laryngeal carcinoma was higher than that in CD133- laryngeal carcinoma cells [50–53] (Figure 1). However, the mechanism of cancer chemo-radioresistance needs further study.

#### GLUT-1 in head and neck cancer

Our previous studies showed high expression of GLUT-1 in head and neck cancer [54], ceruminous adenoma [55], head and neck inflammatory myofibroblastic tumors [56], and nasal and laryngeal neuroendocrine carcinoma [57]. Li detected GLUT-1 expression in all 25 cases of head and neck cancer examined, with expression found mainly in the cell membrane. Poorly differentiated non-keratinizing squamous cell carcinoma cells of the head and neck were located in the central and peripheral nests, while differentiated keratinizing squamous cell carcinoma cells of the head and neck cancer were found in the nest. In addition, the GLUT-1 staining index (SI) was significantly higher in recurrent than in primary head and neck squamous cell carcinoma (*P*=0.03); the GLUT-1 SI was significantly lower in well-differentiated than in poorly differentiated head and neck squamous cell carcinoma (*P*=0.02) [58]. However, GLUT-1 expression in different head and neck cancers showed varying relationships with parameters such as progression and prognosis. These differences are reviewed as follows.

#### Salivary gland tumors

Only a few reports have focused on the clinical significance of GLUT-1 expression in salivary gland tumors. These studies demonstrated that GLUT-1 expression was related to the biological behavior of salivary gland tumors.

#### Features of GLUT-1 expression in salivary tumors

GLUT-1 expression was not detected in normal salivary gland tissues but wasfound mainly in the cytoplasm of pleomorphic adenomas (PAs), adenoid cystic carcinomas (ACCs), and mucoepidermoid carcinomas (MECs) of the salivary glands by de Souza et al. (2017) [59]. Some cases showed membrane staining only in focal areas. The intensity of immunostaining in the squamous metaplasia areas of PAs, epidermoid cells of MECs, and necrotic areas of ACCs was significantly higher than that in other areas. These findings were also observed by Mori et al. [60] and Bonfitto et al [61]. However, Demasi et al. detected GLUT-1 expression mainly in the cell membrane of higher grade MECs [62].

#### GLUT-1 may be related to salivary tumor progression

Mori et al. detected higher expression of GLUT-1 mRNA in ACC than in normal salivary glands by RT-PCR [60]. In our previous study, GLUT-1 mRNA and protein showed high expression in ACC-2 cell lines [63]. Demasi et al. found higher expression of GLUT-1 in high-grade MEC than in low-grade MEC of the salivary gland as detected by IHC. They suggested GLUT-1 to be a potential prognostic factor for MEC of the salivary gland. The possible mechanism of GLUT-1 overexpression in some intermediate- and in all high-grade MECs could require a greater energy supply by acquiring the bioenergetic phenotype of increased glycolysis, leading to the outgrowth of MEC cells under hypoxia [62]. Kim et al. (2011) detected GLUT-1 expression in 17 carcinoma ex PAs (CXPAs) of the salivary glands by IHC, with higher expression in the carcinoma components of CXPAs with luminal differentiation than in residual PA components (*P*=0.024) [64].

#### GLUT-1 may be related to ^18^F-fluorodeoxyglucose (FDG) uptake

In the study by Kim et al. (2011), 6 of 17 patients underwent ^18^F-FDG positron emission tomography (^18^F-FDG PET) [64], and a relationship between high ^18^F-FDG uptake and GLUT-1 overexpression was revealed [64].

#### GLUT-1 may be a poor prognostic factor of salivary tumors

Mori et al. used a Cox proportional hazard model to show that GLUT-1 expression (hazard ratio: 4.739; *P*=0.022) and lymph metastasis (hazard ratio: 3.944; *P*=0.042) are significant prognostic factors of malignant salivary tumors. They also found that some clinicopathological parameters, including age (*P*=0.003), tumor size (*P*=0.002), and distant metastasis (*P*=0.007), were significantly correlated with high GLUT-1 expression [60]. The authors suggested the following potential underlying mechanisms: i) overexpression of GLUT-1 leads to a higher glucose supply for malignant salivary tumors to accelerate and progress, ii) hypoxia within malignant salivary tumors are highly invasive, causing metastasis and a poor prognosis, and iii) hypoxia also results in resistance to chemo-radiotherapy [60].

#### Laryngeal carcinoma

#### Features of GLUT-1 expression in laryngeal carcinoma

In our previous study, GLUT-1 immunostaining was diffuse in the membranes of laryngeal cancer cells, with a rate of GLUT-1 positivity of 55.1% [65].

#### Relationship between GLUT-1 overexpression and progression, metastasis, and prognosis of laryngeal carcinoma

Whether the expression of GLUT-1 is a prognostic factor of laryngeal carcinoma remains debatable. In our previous study, we determined the expression of GLUT-1 and GLUT-3 in 38 tissue samples of primary head and neck cancers, including four laryngeal carcinomas, by RT-PCR and Western blotting. We found that high gene and protein expression of GLUT-1, but not GLUT-3, was associated with poor survival [54]. We also investigated the relationship between HIF-1α or GLUT-1 expression and clinicopathological features or prognosis in 49 tissue samples of laryngeal carcinoma. On univariate analysis, GLUT-1 expression was significantly associated with recurrence (*P*=0.02) and metastasis (*P*=0.01). On multivariate analysis, GLUT-1 expression (*P*=0.006), the primary site in supraglottic and subglottic areas (*P*=0.038), lymph node metastasis (*P*=0.007), and distant metastasis were associated with poor survival [65]. Starska et al. (2015) found that the GLUT-1 mRNA level was significantly associated with the degree of histological differentiation (*P*=0.0005), tumor grade (*P*<0.001), and 5-year survival rate (*P*=0.012) in 106 laryngeal squamous cell carcinoma cases [66]. However, GLUT-1 and GLUT-3 levels were not associated with prognosis. In their study, a worse overall survival rate was found in cases with high GLUT-1 and GLUT-3 expression, but this was only a trend. At the protein level, higher-grade tumors exhibited higher expression of GLUT-1 [66]. Zuo et al. (2016) evaluated whether hypoxia enhanced the invasion and migration of laryngeal carcinoma *in vitro* and investigated the relationships between GLUT-1 expression and biological behavior and prognosis in 57 tissue samples of laryngeal carcinoma [67]. They found that 85.96% of the laryngeal carcinoma samples were positive for GLUT-1, whereas 8.0% of normal laryngeal squamous epithelial tissue and 21.05% of laryngeal carcinoma adjacent tissues were positive for GLUT-1 (*P*<0.05). GLUT-1 overexpression was associated with clinical stage, lymph node metastasis, and poor prognosis. GLUT-1 expression also associated with the expression of epithelial-mesenchymal transition (EMT)-related proteins [67]. Furthermore, molecular mechanism studies showed that hypoxia enhanced laryngeal carcinoma SCC10A cell invasion and migration via EMT [67]. However, Kwon et al. (2014) examined the predictive values of HIF-1α, carbonic anhydrase IX (CAIX), GLUT-1, cyclooxygenase-2, Ki-67, and erythropoietin receptor in 42 tissue samples of T1/T2 early-stage laryngeal carcinomas treated with radiotherapy alone by tissue microarray analysis and IHC and found that only high CAIX was significantly associated with residual tumors on multivariate analysis [68]. Schrijvers et al. (2008) detected three hypoxia markers, HIF-1α, CAIX, and GLUT-1, in 91 tissue samples of T1/T2 glottic carcinoma by IHC and found no significant relationship between GLUT-1 and HIF-1α expression, and GLUT-1 overexpression was not a predictor of worse local control or prognosis [69]. In another study, we investigated the expression of HIF-1α, GLUT-1, and proteins of the PI3K/Akt pathway in 24 tissue samples from patients with laryngeal carcinoma who received PET/computed tomography (CT) preoperatively [70]. However, we found that the maximum SUV (SUV_max_) (*P*=0.043) and PI3K (*P*=0.012) were significantly poor prognostic factors, and that GLUT-1, HIF-1α, and p-Akt were not correlated with prognosis, recurrence, or metastasis [70].

We suggest that the differential findings regarding the relationship between GLUT-1 expression and prognosis of laryngeal carcinoma may be attributed to differences in histopathological type, IHC technique, tumor stage, and sample number, among studies.

#### Relationship between the overexpression of GLUT-1 and ^18^F-fluoroazomycinarabinoside (^18^F-FAZA) or FDG uptake

Bruine de Bruin L (2015) evaluated the relationships of three endogenous hypoxia markers (HIF-1α, CAIX, and GLUT-1) with FAZA uptake in 11 patients with laryngeal carcinoma indicated for total laryngectomy and found positive immunostaining for GLUT-1 in all biopsies but no relationship of any of these three markers with FAZA uptake [71]. In our previous study, we found that ^18^F-FDG uptake (SUV_max_) was associated with GLUT-1 (r=0.577; *P*=0.003), HIF-1α (r=1.0; *P*<0.0001), PI3K (r=1.0; *P*<0.0001) and p-Akt (r=0.577; *P*=0.003) expression [70]. *in vivo*, however, the SUV (tumor/normal tissue) was not associated with GLUT-1 or HIF-1α expression [72].

#### Targeted GLUT-1 expression as a therapeutic strategy for laryngeal carcinoma *in vitro* and *in vivo*

The mechanisms underlying GLUT-1 as a new therapeutic target in laryngeal carcinoma have been discussed in our published review [73]. *In vitro*, we found that antisense oligodeoxynucleotides (AS-ODNs) against GLUT-1 decreased glucose uptake and proliferation of Hep-2 laryngeal carcinoma cells by reducing GLUT-1 mRNA and protein expression [74]. In a previous study, we successfully isolated CD133^+^ cancer stem cells (CSCs) from Hep-2 cells and found higher GLUT-1 mRNA and protein expression in CD133^+^ than in CD133^-^ Hep-2 cells. The proliferation of CD133^+^ Hep-2 cells was higher than that of CD133^-^ Hep-2 cells. These results suggest that GLUT-1 is the main energy supply for laryngeal CD133^+^ Hep-2 cells and is a potential therapeutic target for inhibition of laryngeal CSC proliferation [50]. Our subsequent studies demonstrated that inhibition of GLUT-1 expression in laryngeal carcinoma cells may enhance the chemo-radiosensitivity of laryngeal carcinoma. In a previous study, resistance or insensitivity of Hep-2 cells to cisplatin was associated with GLUT-1 expression. Apigenin, a natural phytoestrogen flavonoid present in various fruits, vegetables (especially celery), beans, and tea, significantly inhibited the expression of GLUT-1 and p-Akt expression to improve the sensitivity of Hep-2 cells to cisplatin [75]. *In vitro* and *in vivo*, we found that the proliferation of Hep-2 cells did not increase with an increasing X-ray dose, and this accorded with the change in GLUT-1 mRNA expression in Hep-2 cells observed with different X-ray radiation doses. We suggest that GLUT-1 expression plays a role in the radioresistance of Hep-2 cells. Inhibition of GLUT-1 expression in Hep-2 cells via AS-ODNs may gradually decrease survival rate and increase the apoptotic rate with prolonged culture time and increased radiation *in vitro*. *In vivo*, the weight of tumors treated with 8 Gy radiation and GLUT-1 AS-ODNs was significantly lower than that after 8 Gy radiation alone. The underlying mechanism was the AS-ODN-induced decrease in GLUT-1 expression [51]. Another mechanistic study of laryngeal carcinoma radioresistance revealed that the PI3K/Akt pathway is involved, mediated by GLUT-1 overexpression [53]. Apigenin may enhance laryngeal carcinoma radiosensitivity through the inhibition of GLUT-1 expression and the PI3K/Akt pathway *in vivo* [52]. We first demonstrated co-inhibition of GLUT-1 expression via AS-ODNs and the PI3K/Akt pathway via specific inhibitors including Ly294002 and wortmannin. After 10 Gy X-ray radiation, Ly294002, wortmannin, Ly294002 plus GLUT-1 AS-ODNs, and wortmannin plus GLUT-1 AS-ODNs reduced the tumor size significantly compared with tumors treated with 10 Gy X-ray radiation only (*P*<0.05). Similarly, the expression levels of GLUT-1, p-Akt, and PI3K were significantly decreased by GLUT-1 AS-ODNs and PI3K/Akt inhibitors [53]. The mechanism of laryngeal carcinoma radioresistance may involve multiple factors. In our recent study using Hep-2 and Tu212 laryngeal carcinoma cells, 10 Gy X-ray radiation decreased the weight of Hep-2 and Tu212 xenografts, while GLUT-1 AS-ODNs decreased the weight of Tu212 xenografts only. Combined with HIF-1α, GLUT-1 AS-ODNs may significantly increase apoptosis and decrease microvessel density, the apoptotic index, and necrosis after X-ray irradiation *in vivo* [72].

#### Oral carcinoma

#### Expression of GLUT-1 in oral carcinoma

GLUT-1 expression was confirmed in 100% of 50 cases of oral squamous cell carcinoma by IHC staining [76]. Pereira et al. (2016) detected GLUT-1 in 15 samples from patients with oral epithelial dysplasia (OED) and 15 samples from patients with oral squamous cell carcinoma (OSCC) by IHC. GLUT-1 expression was positive in all cases of OED and OSCC. GLUT-1 immunostaining was greater in OED than that in OSCC, suggesting that GLUT-1 is expressed during the initial stages of oral carcinoma [77]. Leite et al. (2017) detected GLUT-1 and GLUT-3 expression in both keratocystic odontogenic tumors associated with Gorlin syndrome (SKOTs) and non-syndromic keratocystic odontogenic tumors (NSKOTs) by IHC. They revealed positive GLUT-1 expression in the epithelial component in all cases [78]. They found that GLUT-1 and GLUT-3 were not associated with the angiogenic index in SKOTs, primary NSKOTs, or recurrent NSKOTs [78].

#### Relationship among GLUT-1, differentiation of oral carcinoma, and cellular distribution

Azad et al. found that the expression of GLUT-1 in oral squamous cell carcinoma was also closely related to smoking history (*P*<0.001), Bryne grade (*P*<0.001), tumor size (*P*=0.001), lymph node metastasis (*P*=0.022), and clinical stage (*P*<0.001).GLUT-1 also shows a progressive switch from membranous to cytoplasmic to a combined location and is correlated with histopathologic grade and pTNM stage. As tumors become more malignant, higher levels of GLUT-1 accumulate at the cell membrane to allow transport of glucose into cells, so that tumors can proliferate rapidly [76]. That report suggested that mature squamous epithelium is associated with glycogen accumulation during tumor formation. The presence of glycogen is related to the maturation of squamous epithelial cells, in that it disappears with the loss of differentiation during neoplastic transformation. In well-differentiated tumors, increased accumulation of glycogen in keratin pearls is inversely correlated with GLUT-1 immunostaining, suggesting that differentiated and mature cells within keratinized regions lack GLUT-1 expression. In contrast to poorly differentiated tumors, hypoxia-stimulated GLUT-1 creates an antistromal staining pattern in the absence of squamous epithelial differentiation or keratinization [76]. Among 57 cases of tongue squamous cell carcinoma, 52.6% had positive GLUT-1 expression in the tumor periphery and 47.4% in the tumor center, and this was associated with the classification of the tongue cancer tissue. In advanced tongue squamous cell carcinoma, 90.7% of samples were strongly positive for GLUT-1, with low levels of GLUT-1 in tumor cells in the tumor periphery and high levels in the central tumor [79]. A recent interesting study investigated the role of oral brush biopsy and GLUT-1 staining in 72 patients, including 24 healthy patients, 15 with carcinoma, 18 with leukoplakia, and 15 with oral lichen planus. The results showed positive GLUT-1 expression in 30 (41.7%) patients, including 80% (12) with HNSCC, 60% (9) with oral lichen planus, and 50% (9) with leukoplakia. No GLUT-1 staining was found in the healthy patients [80]. The authors suggested that GLUT-1 overexpression is likely an early event in carcinogenesis with prognostic and therapeutic value [80].

#### GLUT-1 and TNM stage of oral carcinoma

Ohba et al. found that GLUT-1 expression was related to the depth of oral cancer invasion. A significant difference was found in GLUT-1 staining intensity between tumors with < 4 and those with > 4 mm invasion depth (*P*=0.023), indicating that GLUT-1 expression is a marker of the extent of tumor invasion [81].

#### GLUT-1 expression and prognosis of oral carcinoma

Ayala et al. found that GLUT-1 was highly expressed in 142 patients with oral squamous cell carcinoma, while GLUT-3 was highly expressed in 21.1% of these patients, and high protein expression of GLUT-1 and GLUT-3 was associated with poor prognosis [82]. Studies have found that GLUT-1 expression alone cannot be used as an independent prognostic marker for head and neck cancer. Grimm found that increased expression levels of both GLUT-1 and transketolase-like protein 1 are markers of poor prognosis in oral cancer [83]. Eckert found that high expression levels of both GLUT-1 and HIF-1a are markers of poor prognosis in oral cancer [84].

#### Thyroid cancer

#### GLUT-1 expression

GLUT-1 expression in thyroid cancer is controversial. *In vitro*, GLUT-1 expression is significantly higher in anaplastic thyroid cancer cells than in normal cells [85]. Musholt et al. (1997) detected GLUT-1–5 in 10 medullary thyroid carcinoma (MTC) samples by Western blotting, one of which exhibited very weak GLUT-1 expression [86]. Kaida et al. (2011) evaluated the relationship between ^18^F-FDG uptake and GLUT expression or clinicopathological factors in 54 patients with papillary thyroid cancer (PTC). The results revealed positive expression of GLUT-1, GLUT-3, and GLUT-4 in the cytoplasm and/or membrane of PTC cells [87]. GLUT-3 and GLUT-4 expression was stronger than GLUT-1 expression. A GLUT-1 expression score of 3 was found in only one tumor with anaplastic features, suggesting that strong GLUT-1 expression may occur only in PTC with anaplasia [87]. Among 10 patients with thyroid tissue showing thymus-like elements (CASTLE), GLUT-1 expression was positive in all [88]. The authors suggested that GLUT-1 is a novel biomarker for CASTLE with potential diagnostic value [88]. Kim et al. (2013) detected a rate of GLUT-1 positivity of 66.7% in 188 patients with PTC who underwent ^18^F-FDG-PET/CT examination [89]. However, Chandan et al. (2006) detected CD57 and GLUT-1 expression in 50 thyroid fine-needle aspiration samples, including 15 papillary carcinoma, 14 atypical cytology, and 21 benign thyroid cases. They found that 20 of 29 cases with malignant lesions were positive, while all 21 benign thyroid cases were negative, for CD57 expression; however, GLUT-1 expression was negative in all 50 malignant and benign thyroid lesions [90].

#### GLUT-1 expression, biological behavior, and prognosis of thyroid cancer

Kim et al. (2014) investigated the expression of GLUT-1 and major thyroid-specific genes in 24 PTC tissue samples by RT-PCR [92]. They detected higher GLUT-1 expression in the less-differentiated group than in the well-differentiated group among 23 cases [91]. Lodewijk et al. (2017) detected HIF-1α, CAIX, and GLUT-1 expression in 111 MTC tissue samples by IHC. On univariate analysis, HIF-1α was associated with overall survival and progression-free survival (PFS), and GLUT-1 was significantly associated with PFS, as well as TNM stage, the presence of lymph node metastasis, heritability, and necrosis. However, only HIF-1α, TNM stage, and heritability were prognostic factors on Cox regression analysis (r=0.21; *P*=0.147) [92]. Kaida et al. (2011) found that the prognosis of PTC was not correlated with age, sex, extrathyroid extension, SUVmax, or GLUT-1, GLUT-3, or GLUT-4 expression [87]. Kim et al. (2013) found that GLUT-1 expression was not associated with SUV_max_, extrathyroidal extension, lymph node metastasis, or advanced tumor stage in patients with primary thyroid cancer [93].

#### ^18^F-FDG uptake

The findings of Kaida et al. (2011) showed that the SUV_max_ was associated with GLUT-3 (r=0.38; *P*=0.008) and GLUT-4 (r=0.46; *P*=0.001) but not GLUT-1 expression (r=0.21; *P*=0.147) [87]. Among 38 patients with thyroid papillary cancer with recurrent cervical nodal metastases, 21 (55%) exhibited positive ^18^F-FDG uptake, which was related to thyroid globulin expression and GLUT-1 membrane expression with luminal accentuation. However, ^18^F-FDG uptake was not associated with GLUT-1 cytoplasmic expression [94]. Kim et al. (2013) found that ^18^F-FDG uptake was not associated with GLUT-1 or HIF-1α expression [89].

### HK-II

#### HK-II biological characteristics

HK is a key enzyme in glucose metabolism [95]. Mammals have four isoforms of HK (HKs I–IV). HK isoforms exhibit some organizational differences and specific distribution patterns *in vivo* [96]. HK-I is expressed mainly in the brain, HK-II mainly in insulin-sensitive tissue such as myocardial and skeletal muscle and adipose tissue, HK-III in kidney, liver, and intestinal tissues, and HK-IV in the liver and pancreas. When glucose enters the cell, the first step is its phosphorylation into glucose-6 phosphate, which is not able to cross the cell membrane. The first key rate-limiting enzyme in this process is HK [96]. In normal tissue, free HK molecules are predominant. However, in tumor tissues, HK can combine with mitochondria, forming particles of HK, of which the N-terminal domain has a hydrophobic end connected to the outer mitochondrial membrane; this then forms a complex with the mitochondrial permeability tunnel complex of the voltage dependent anion channel protein (VDAC) binding to HK and forming HK-VDAC [51]. HK-VDAC can enhance the ability of ATP to bind to mitochondria and to supply tumor cells with energy. HK-VDAC is also a major contributor to the immortalization of cancer cells [97].

Studies have shown that the disruption of HK-VDAC can lead to apoptosis via the PI3K/Akt signaling pathway [98]. However, the high levels of lactic acid produced by glycolysis can help tumor cells escape from immune detection and allow their rapid proliferation [96]. The four subtypes of HK (I-IV) are highly expressed in malignant tumors, with HK-II being the most highly expressed, and the proportion of HK-IIb in microparticles is higher than that of the other subtypes [97].

#### HK-II and malignant tumors

#### HK-II expression in malignant tumors

Several research reports have shown increased HK-II expression in many malignant tumors, including nasopharyngeal cancer, ovarian cancer, renal cell carcinoma, hepatocellular carcinoma, colon cancer, and glioma [98–100]. A five-fold increase in the gene expression of HK-II, but not the other HK isoforms, was detected in liver tumors, and this is thought to accelerate glycolysis in hepatoma cells to provide extra energy [97]. Guzman et al. found that the expression of HK-II was significantly higher in hepatocellular carcinoma than in the control group, and that the high HK-II expression was correlated with invasiveness and high tumor grade [100]. Wolf et al. found that in 25 patients with pleomorphic gliomas of the brain, 20 showed HK-II expression in the brain but not in normal human brain white matter [101].

The expression level of HK-II is 200-fold higher in malignant tumor tissues than in normal tissues. Moreover, it was found that the rate of glycolysis in hepatocytes was significantly increased after the introduction of mitochondrial binding HK-II [102]. Guzman also found that the expression of HK-II increased incrementally from normal liver tissue to the compensated and decompensated stages of liver cirrhosis to the development of liver cancer [100]. This pattern would suggest that HK-II increases during tumor development from normal tissue to precancerous lesions, playing an important role in tumor development.

#### The relationships between HK-II and the clinical stage, differentiation, metastasis, and prognosis of malignant tumors

HK-II was found to be related to the clinical stage, differentiation, metastasis, and poor prognosis of malignant tumors [103–108]. Hamabe et al. analyzed 104 cases of colorectal cancer by IHC, dividing the samples into HK-II expression-positive and -negative groups. They found that the expression of HK-II was related to tumor diameter (*P*=0.046), depth of tumor invasion (*P*=0.0395) and lymph node metastasis (*P*=0.0409) in the HK-II-positive group [83]. Jin et al. found that HK-II expression was significantly higher in phase III/IV than phase III ovarian cancer (*P*<0.001), and HK-II expression was significantly higher in the poorly differentiated than the highly differentiated group (*P*=0.008) [98]. Other reports have shown that the expression of HK-II in phase II to IV was significantly higher than that in phase I cancer (*P*=0.044) [100]. The high expression of HK-II in colorectal cancer was related to tumor size, depth of tumor invasion, liver metastasis, recurrence, and TNM stage. Multivariate analysis showed that HK-II was an independent prognostic factor for colorectal cancer [109]. Similar results were found in liver cancer [103], pancreatic cancer [110], cervical cancer [111], gastric cancer [106], breast cancer [112], and lung cancer [113].

#### HK-II in head and neck cancer

To our knowledge, few studies have focused on HK-II in head and neck cancer. Thus, this section has not been subdivided according to different tissues and cell types of head and neck tumors.

#### The relationship between HK-II and head and neck cancer

Chen et al. found that the expression of HK-II in laryngeal carcinoma tissue, laryngeal papilloma, and laryngeal polyps was 100%, 37%, and 10%, respectively [114]. They suggested that the expression of HK-II was related to the occurrence and progression of laryngeal carcinoma [114]. Tian et al. detected HK-II expression in all 19 cases of oral squamous cell carcinoma examined [115].

#### The relationship between HK-II and the stage, differentiation, metastasis, and prognosis of head and neck cancer

Chen et al. found that the expression of HK-II was associated with the tumor site and TNM stage of laryngeal carcinoma [114]. A possible mechanism may be that glucose phosphorylation and HK-II binding to the mitochondrial membrane were increased, which inhibited cytochrome C release and apoptosis and reduced glucose-6-phosphate negative feedback on glycolysis; this may have increased glycolysis and substrate product biosynthesis, increasing the growth of cancer cells and acidulating the cancer microenvironment to promote tumor invasion [114].

Xiao et al. performed a retrospective analysis of 41 patients with nasopharyngeal carcinoma who received radiotherapy or chemoradiotherapy. They found that the median survival of 21 patients with high HK-II expression was 62.29 months, and that of 20 patients with low HK-II expression was 93.60 months. The high expression of HK-II was associated with prognosis. It was also found that inhibition of HK-II expression enhanced the radiosensitivity of nasopharyngeal carcinoma [116].

#### Possible mechanism of radioresistance caused by HK-II in cancer

The underlying mechanism of HK-II-induced radioresistance in laryngeal carcinoma has been described in our previous review. HIF-1α, suppressor oncogenes, miR-155 may involve in the mechanisms of radioresistance.

### GLUT-1 and HK-II

#### GLUT-1, HK-II, and the Warburg effect

The Warburg effect has been confirmed by uptake of ^18^F-FDG), an analogue of glucose, on PET/CT. ^18^F-FDG is transported into the cell by GLUT after intravenous injection and is phosphorylated by HK to generate ^18^F-FDG -6-phosphate, which is not further metabolized and thus deposited in cells, showing a high metabolic concentration on PET images [117].

#### Expression of GLUT-1 and HK-II in malignant tumors and their correlation with FDG uptake

Several studies have shown that GLUT-1 and HK-II expression levels are related to tumor FDG uptake, but there are differences among tumor types. The uptake of FDG by some malignant tumors was related to tumor expression levels of GLUT-1 or HK-II, or both; however, uptake of FDG by other tumors was independent of either GLUT-1 or HK-II expression [120]. Therefore, the mechanism of FDG uptake in malignant tumors requires further study.

Several studies have reported a relationship between GLUT-1 and HK-II expression in malignant tumors [119, 120]. The rapid uptake of glucose via GLUT-1 in cancer cells is maintained mainly by HK-II. Grimm et al. found that GLUT-1 and HK-II expression significantly increased with the progression from normal oral mucosa, dysplasia, and neoplasia at stages I, II, and III to invasive squamous cell carcinoma [119]. The expression of GLUT-1 in oral squamous cell carcinoma was nearly four-fold higher than that in normal tissues [119]. Pauday et al. found that GLUT-1 expression was associated with HK-II expression in hepatobiliary cancer cells (*P*=0.002, *P*=0.3), and FDG uptake was also associated with GLUT-1 or HK-II expression [120]. In the breast cancer cells of nude mice, Kristian et al. used dynamic PET/CT to show that a high initial FDG uptake was positively correlated with increased expression of GLUT-1, and FDG uptake during the late phase was associated with HK-II expression. Therefore, GLUT-1 and HK-II may play roles during different periods of FDG uptake [121]. Cancer cells significantly increase their demand for glucose as malignancy progresses, and GLUT-1 and HK-II simultaneously increase in expression, which is associated with the SUV. These studies suggest that GLUT-1 and HK-II play a synergistic role in FDG uptake during the progression of malignant tumors.

Inconsistent results were reported for some malignant tumors, however. While HK-II was associated with GLUT-1 expression during stages T3 and T4 of oral squamous cell carcinoma (*R*=0.99, *P*=0.0001), there was no correlation between the expression of HK-II and FDG uptake [115]. In rectal cancer, Izuishi et al. found that GLUT-1 and HK-II expression was significantly higher in cancerous tissue than in normal tissue surrounding the mucosa, whereas FDG uptake was not associated with GLUT-1 or HK-II expression. They suggested that aberrant glucose-metabolizing pathways in cancer may not be regulated by a single molecule [122]. Although Yoon et al. found that GLUT-1 and HK-II expression levels were as high as 81% and 77%, respectively, in extrahepatic bile duct carcinoma, there were no correlation between their expression levels [4]. The SUV or SUV tumor-to-liver ratio was associated with GLUT-1 expression (ρ=0.648, *P*=0.0003 and ρ=0.703, *P*<0.0001, respectively), but not with HK-II expression [4]. Yang et al. detected the expression of HK-II and GLUT-1 using IHC in 50 patients with pancreatic cancer who underwent preoperative PET/CT and found that the expression of GLUT-1 and HK-II in cancer tissues was higher compared with the adjacent tissue (*P*<0.001). However, there was no correlation between FDG uptake and the expression of GLUT-1 or HK-II or between GLUT-1 and HK-II [118]. Higashi et al. also found a high correlation between GLUT-1 and HK-II expression in pancreatic cancer but no association with FDG uptake (*P*=0.055 and *P*=0.1852, respectively) [123]. Cho et al. found that GLUT-1 and HK-I expression levels were increased with higher grades of gastrointestinal stromal tumor risk. However, HK-II and several other markers (i.e., GLUT-2, -3, and -4) were not correlated with tumor risk grade. FDG absorption was related to the expression of GLUT-1 and HK-I [117]. Baschnagel et al. found that the expression of HK-II was correlated with the epidermal growth factor receptor, but not with p16 or GLUT-1, in 97 patients with locally advanced head and neck squamous cell carcinoma. The SUVmax was associated with HK-II expression (*P*=0.021), but not GLUT-1 expression [124]. Zhou et al. found that FDG uptake was associated with the expression of GLUT-1 and lactate dehydrogenase (LDHA), but not HK-II, in 51 cases of lung adenocarcinoma. *In vitro*, they found that knockdown of LDHA can reduce FDG uptake, GLUT-1 expression, and cell proliferation [125].

Park et al. found an absence of HK-II expression in 19 cases of malignant melanoma, and FDG uptake was correlated with the expression of GLUT-1 and GLUT-3, mainly GLUT-1 [126]. In five different breast cancer xenograft models, HK-II protein expression was significantly related to FDG absorption (r^2^=0.339, *P*=0.001), and HK-II was an independent predictor of FDG uptake. There was a relationship between GLUT-1 and FDG uptake only in tumors driven by Akt or the HER2/neu gene [127]. Takebayashi et al. reported high expression of HK-I and HK-II in 50 cases of gastric carcinoma; however, no relationship was detected between FDG uptake and GLUT-1 or HK-II expression [128].

These results suggest that in some malignant tumors, GLUT-1 and HK-II are not the only critical rate-limiting enzymes, and that HK-II expression is not completely consistent with GLUT-1 expression. Yang et al. suggested that glucose metabolism is a complex process involving multiple factors, including GLUT-2, -3, and -4 and HK-I; however, the actual mechanism requires further study [118].

### The effect of hypoxia on the expression of GLUT-1 and HK-II in head and neck cancer

Hypoxia is a key regulator of the expression of GLUT-1 and HK-II. Malignant tumors, including head and neck cancers, arise in hypoxic environments [66]. Hypoxia can alter tumor cells physiologically and biochemically by regulating the expression of a variety of target genes, including GLUT-1 and HK-II, involved in adaption to the hypoxic environment.

HIF-1α plays an important role in the adaptive cellular response to hypoxia [26]. Co-expression of GLUT-1, HK-II, and HIF-1α has been observed in numerous tumor types [42, 129, 130]. HIF-1α can activate the expression of target genes such as GLUT-1, HK-II, and VEGF [26, 42, 129, 130].

*In vitro*, Iwamoto et al. found that ^18^F-FDG was significantly accumulated in KRAS-mutated cells compared with wild-type cells under normoxic conditions, and that GLUT-1 and HK-II mRNA levels were high. FDG uptake was associated mainly with GLUT-1 expression. A possible mechanism may be that the Raf/MEK/ERK pathway is correlated with GLUT-1 expression in KRAS-mutated cells, while the PI3K/Akt pathway is correlated with HK-II expression in both KRAS-mutated and wild-type cells [131]. Under hypoxia, the expression of HIF-1α was higher in KRAS-mutated cells compared with wild-type cells. Overexpression of HIF-1α resulted in higher GLUT-1 expression and ^18^F-FDG accumulation. That same study showed that increased FDG uptake was related mainly to GLUT-1 expression *in vivo* [131]. In acute myeloid leukemia, HIF-1α, GLUT-1, and HK-II were highly expressed [132]. HIF-1, GLUT-1, and HK-II were also highly expressed in 36 cases of stage T1 and T2 squamous cell carcinoma of the oral cavity and were significantly correlated with FDG uptake; furthermore, the expression of HIF-1 was related to the expression of GLUT-1 and HK-II [133]. Kim et al. (2017) detected glycolysis markers, including GLUT-1, HK-II, CAIX, and MCT4, in 265 follicular neoplasm (FN) samples and 108 Hürthle cell neoplasm (HCN) samples by tissue microarray and IHC staining and found that these markers were highly expressed in HCNs compared with in FNs, with the highest expression in Hürthle cell carcinoma (HCC) followed by Hürthle cell adenoma, follicular carcinoma (FC) and follicular adenoma (FA), in that order (all *P*<0.001). HK-II expression was correlated with a larger HCC tumor size (> 4 cm) (r=0.384; *P*=0.046). Expression of the Ki67 proliferation index was related to GLUT-1 expression in FC (r=0.419; *P*=0.029). In this study, however, the expression of the glycolysis markers GLUT-1, HK-II, CAIX, and MCT4 was not significantly related to the prognosis of FC or HCC on multivariate analysis [134].

### GLUT-1, HK-II, and tumor-targeting therapy

**Figure 2:**
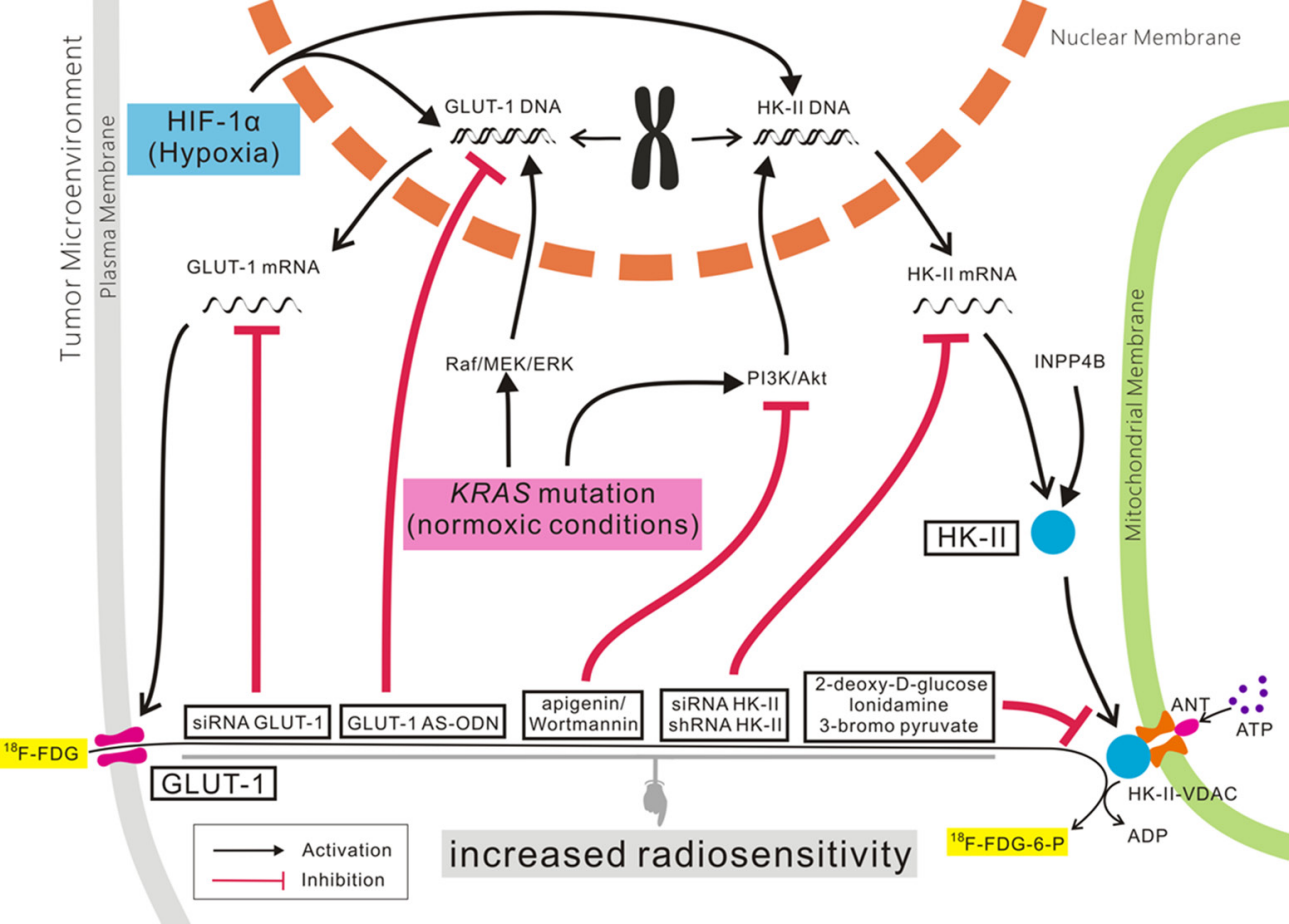
The role of GLUT-1 and HK-II expression in cancer.

As mentioned above, the development, metastasis, and poor prognosis of some malignant tumors were correlated with abnormal expression of GLUT-1 and HK-II. Many studies have found that GLUT-1 and HK-II expression is related to chemo- and radioresistance in some malignant tumors, and GLUT-1 and/or HK-II may represent anti-cancer therapeutic targets (Figure 2) [73, 95].

Our previous studies found that high expression of GLUT-1 was associated with radioresistance in laryngeal carcinoma, and that inhibition of GLUT-1 expression by antisense oligodeoxynucleotides (AS-ODNs) may improve the radiosensitivity of laryngeal carcinoma, *in vitro* and *in vivo* [51, 74]. *In vivo*, apigenin/wortmannin or apigenin/wortmannin plus GLUT-1 AS-ODNs improved the radiosensitivity of laryngeal carcinoma [52, 53].

Similar results have been reported in other malignant tumors [135–137]. A GLUT-1-specific AS-ODN or siRNA, combined with inhibition of the PI3K/Akt signaling pathway, can improve the radiosensitivity of laryngeal cancer cells to some degree. However, this effect was found mainly *in vitro*, with unsatisfactory results *in vivo*. Therefore, some authors have suggested that it may be more effective to improve radiosensitivity via combined inhibition of GLUT-1 and other proteins involved in the Warburg effect, such as HIF-1 or HK-II.

As mentioned above, high expression of HK-II is associated with chemo- and radioresistance [138–145]. In recent years, research has increasingly focused on HK-II as a target for anti-tumor therapies, e.g., 2-deoxy-D-glucose [141], lonidamine [142], 3-bromo pyruvate [143]. Inhibition of HK-II expression in cancer cells using a targeted HK-II-VDAC complex [144] and siRNAs [145] induced apoptosis of cancer cells.

Chen et al. found that high expression of HK-II was related to the TNM stage of laryngeal carcinoma [114]. It was found *in vivo* and *in vitro* that inhibition of HK-II expression by shRNAs inhibited the growth of laryngeal carcinoma [114]. Min et al. found that inositol polyphosphate 4-phosphatase (INPP4B) can regulate HK-II-induced glycolysis, leading to radioresistance in laryngeal carcinoma. Inhibition of HK-II expression alone did not improve the sensitivity to radiotherapy and chemotherapy in laryngeal carcinoma cells, while co-inhibition of INPP4B and HK-II did [138].

## CONCLUSIONS AND THERAPEUTIC PERSPECTIVES

These results suggest that simultaneous inhibition of HK-II and glycolysis-related genes may improve the radiosensitivity of laryngeal carcinoma. GLUT-1 and HK-II play important roles in different processes of glycolysis in malignant tumors and may be associated with radioresistance. Inhibition of GLUT-1 or HK-II can increase radiosensitivity to some extent. Therefore, we propose that combined inhibition of GLUT-1 and HK-II expression may enhance tumor chemo- and radiosensitivity and provide new therapeutic targets for malignant tumors.

## Abbreviations

GLUT-1: Glucose transporter 1
HK-II: hexokinase-II
HIF-1α: hypoxia inducible factor-1α
CA-IX: carbonic anhydrase-IX
VDAC: voltage dependent anion channel
PET: protein, positron emission computed tomography
LDHA: lactate dehydrogenase
AS-ODN: antisense oligodeoxynucleotides
3-BrPA: 3-bromo pyruvate
INPP4B: Inositol polyphosphate 4-phosphatase)
